# Proteomic analysis of corneal astigmatism identifies reduced apolipoprotein A-IV as a candidate biomarker

**DOI:** 10.3389/fmed.2026.1761572

**Published:** 2026-07-15

**Authors:** Ximin Wei, Weitao Li, Wei Zhang

**Affiliations:** 1 Center of Ophthalmic Refraction, 3201 Hospital, Hanzhong, Shaanxi, China; 2 Microbiology and Immunology Laboratory, 3201 Hospital, Hanzhong, Shaanxi, China

**Keywords:** apolipoprotein A-IV, biomarker, corneal astigmatism, parallel reaction monitoring, proteomics

## Abstract

**Introduction:**

Astigmatism is a common refractive error associated with myopia, yet its molecular mechanisms remain poorly understood. This study aimed to identify and validate differentially expressed proteins (DEPs) in corneal astigmatism through a label-free quantitative proteomic approach, followed by biomarker validation using parallel reaction monitoring (PRM).

**Methods:**

This two-phase, retrospective case–control study aimed to identify and verify differentially expressed proteins (DEPs) associated with corneal astigmatism. Phase I (discovery) employed label-free quantitative (LFQ) proteomics to compare stromal samples from 9 patients (10 eyes) with compound myopic astigmatism (Group A) and 8 patients (10 eyes) with simple myopia (Group B). Phase II (verification) involved targeted parallel reaction monitoring (PRM) in an independent cohort comprising 13 patients (15 eyes) in Group A and 14 patients (15 eyes) in Group B, quantifying discovery-prioritized proteins. Bioinformatic analyses including COG, GO, KEGG, and protein–protein interaction mapping were performed to characterize enriched pathways and guide PRM target selection.

**Results:**

A total of 127 DEPs were identified, with 31 upregulated and 96 downregulated in Group A. Bioinformatic analysis of the discovery dataset showed enrichment of proteins associated with wound healing, blood coagulation, and lipid metabolism; these enrichments were exploratory and were used only to prioritize candidates for targeted verification. Targeted PRM verified a significant reduction in APOA4 in astigmatism; other candidates showed concordant trends without statistical significance.

**Conclusion:**

After targeted verification, APOA4 was the only protein that remained significantly reduced in corneal astigmatism, supporting reduced APOA4 as a candidate biomarker that warrants further study. The discovery-phase associations with coagulation, lipid metabolism, and wound-healing pathways were not confirmed at the protein level and should be regarded as hypothesis-generating, requiring confirmation in larger, adequately powered cohorts.

## 1 Introduction

Myopia is one of the most prevalent refractive errors worldwide, with its incidence rising rapidly across populations. By 2050, nearly half of the global population is predicted to be affected by myopia, making it a critical public health issue (1). Among individuals with myopia, a significant subset develops astigmatism, causing blurred or distorted vision. While myopia is often managed with corrective lenses or refractive surgery, astigmatism poses additional challenges due to its multifactorial etiology and the complex biomechanical properties of the cornea (2).

Corneal refractive surgery, including small incision lenticule extraction (SMILE), is widely used to correct myopia and astigmatism (3). However, astigmatism remains a key limiting factor in achieving optimal visual outcomes post-surgery. Despite its clinical importance, the molecular mechanisms underlying corneal astigmatism, particularly in the context of myopia, remain poorly understood (4). Addressing this knowledge gap is crucial for improving refractive surgery outcomes and guiding the development of targeted therapies.

Recent advances in proteomics offer powerful tools for characterizing the complex molecular landscape of ocular diseases. Proteomic approaches allow for the comprehensive identification and quantification of proteins, enabling researchers to unravel the intricate biological pathways involved in disease pathogenesis (5–7). In particular, label-free quantitative proteomics has been successfully applied to identify biomarkers and therapeutic targets across a range of ocular conditions, including age-related macular degeneration (8), diabetic retinopathy (8), and keratoconus (9). However, research is yet to apply this approach to investigate the proteomic changes associated with astigmatism in myopic patients.

Recent studies have identified several genes and proteins implicated in corneal curvature and astigmatism. Genome-wide association studies have consistently reported variants near PDGFRA, influencing corneal shape and the risk of astigmatism across multiple populations (10). Structural extracellular-matrix proteins such as lumican (LUM) and decorin (DCN) regulate collagen fibril organization and corneal transparency, and their altered expression has been linked to irregular corneal topography and refractive instability (11, 12). Additionally, oxidative stress-related enzymes including superoxide dismutase-1 (SOD1) and peroxiredoxin-6 (PRDX6) have been associated with corneal remodeling processes and keratoconic changes (13, 14). Despite these insights, evidence integrating proteomic and pathway-level alterations in astigmatic corneas remains scarce, highlighting the need for a comprehensive molecular characterization using high-resolution proteomics.

To fill this gap, we conducted a two-phase proteomic study aimed at identifying and validating differentially expressed proteins (DEPs) between patients with simple myopia and those with compound myopic astigmatism undergoing SMILE surgery. In the first phase, we employed a label-free quantitative proteomic assay to identify DEPs, followed by a bioinformatics analysis to explore the biological processes and pathways implicated in astigmatism. In the second phase, we validated the candidate proteins using parallel reaction monitoring (PRM) to confirm their association with corneal astigmatism.

## 2 Materials and methods

This investigation was a two-phase, retrospective case–control study designed to (i) discover DEPs in corneal stromal tissue by label-free quantitative (LFQ) proteomics and (ii) perform targeted verification of discovery-prioritized proteins using parallel reaction monitoring (PRM). Phase I (discovery) contrasted stromal proteomes from compound myopic astigmatism (case) versus simple myopia (control). Phase II (verification) quantified a prioritized subset in independent targeted runs.

### 2.1 Phase I: exploratory study: proteomic approaches for biomarker discovery

#### 2.1.1 Patient recruitment

This study was approved by the Institutional Review Board of 3,201 Hospital (ethics number 2022046) and adhered to the principles of the World Medical Association Declaration of Helsinki. Twenty-six healthy subjects (30 eyes) aged 18 to 41 years old with generalized myopia were recruited from May to September 2023 who underwent SMILE surgery (VisuMax, Carl Zeiss Meditec, Jena, Germany). All enrolled participants agreed to sample collection and informed consent was obtained. Complete general and ophthalmic medical histories were collected for all participants. The selected eyes were divided into myopia combined with myopic astigmatism group (Group A: 15 eyes in 12 cases) and simple myopia group (Group B: 15 eyes in 14 cases). Corneal curvature was measured with three instruments: a Topcon KR-1 autorefractor (Topcon, Tokyo, Japan), a Pentacam (Oculus, Wetzlar, Germany), which measures both anterior and posterior corneal curvature, and a Zeiss ATLAS 9000 corneal topographer (Carl Zeiss Meditec, Jena, Germany), which measures anterior corneal surface curvature only. Topography obtained with the Pentacam and the ATLAS 9000 was reviewed in every eye to exclude irregular astigmatism. Because there is no single accepted standard and anterior corneal astigmatism is closely correlated with total corneal astigmatism in patients of refractive-surgery age, eyes were required to demonstrate highly consistent astigmatism, defined as an absolute difference of less than 0.50 D between anterior corneal astigmatism (measured by Pentacam or ATLAS 9000) and refractive astigmatism (obtained from manifest refraction). Only eyes meeting this consistency criterion across all three measurements during the collection period were included; consequently, the number of mismatched eyes excluded was not separately tabulated. Group allocation was based on the magnitude of corneal astigmatism: eyes with significant, consistent astigmatism were assigned to the compound myopic astigmatism group (Group A), and eyes without significant astigmatism to the simple myopia group (Group B). To avoid misclassification, no eye with corneal astigmatism greater than 1.0 D was placed in the simple myopia group on the basis of a small refractive astigmatism. Inclusion criteria: Patients undergoing SMILE; No history of ocular trauma or surgery; And the absence of unrelated eye conditions such as cataracts, glaucoma, or retinopathy. Systemic exclusion criteria were the presence of systemic diseases, such as endocrine, respiratory, or cardiovascular disease, renal disease, a serious infectious state, an inflammatory state, or current pregnancy, with no use of any medications (e.g., antimetabolic drugs, immunosuppressants, or steroids).

#### 2.1.2 Sample collection

10 corneal stroma lenticules were randomly selected for each group from the above cohorts. Each corneal stroma sample was stored separately in an Eppendorf tube, numbered on the tube, and frozen in liquid nitrogen immediately after lens removal. The samples were then stored in a refrigerator at − 80 ° C until measurement. Sample preparation, nanoLC-MS/MS analysis, and data/bioinformatics analysis are described in Supplementary File 1.

### 2.2 Phase II: PRM validation of clinical cases

The stage subsequent to 4D-LFQ discovery experiments encompasses a workflow founded on LC-parallel reaction monitoring (PRM) for establishing sensitive and precise detection of the relative abundance of candidate protein markers. From November 2023 to April 2024, a total of 30 corneal stromal lenticules from 28 patients within SMILE procedures were obtained in another clinical cohort, adhering to the identical inclusion and exclusion criteria, including the myopia combined with myopic astigmatism group (Group A: 13 cases, 15 eyes) and the simple myopia group (Group B: 14 cases, 15 eyes). All enrolled participants agreed to sample collection and informed consent was obtained. The limitation of reagent costs demands further prioritization in profiling all identified candidate proteins. Subsequently, 20 validation proteins were comprehensively selected based on the FC value, *p*-value, hierarchical clustering, GO functional enrichment, KEGG pathway enrichment and PPI network analysis results of DEPs in the first stage of label-free proteomics analysis experiment.

#### 2.2.1 Protein extraction and nanoLC-MS/MS analysis

The protein was extracted from 30 corneal stromal validation samples by employing the identical method. The protein concentration was measured by BCA, and the protein integrity was confirmed through SDS-PAGE analysis. The qualified protein samples underwent reductive alkylation, and the polypeptide samples were obtained by desalting after the enzymolysis of Trypsin. For each sample, 2 𝜇g of total peptides were separated and analyzed with a nano-UPLC (nanoElute2) coupled to a timsTOF Pro2 instrument (Bruker) with a nano-electrospray ion source after obtaining polypeptide samples using the same method as LFQ. Separation was performed using a reversed-phase column (PePSep C18, 1.9 𝜇m, 75 𝜇m × 15 cm, Bruker, Germany). Mobile phases were H2O with 0.1% FA (phase A) and ACN with 0.1% FA (phase B). Separation of sample was executed with a 60 min gradient at 300 nL/min flow rate. Gradient B: 2% for 0 min, 2–22% for 45 min, 22–37% for 5 min, 37–80% for 5 min, 80% for 5 min. The mass spectrometer adopts DDA PaSEF mode for DDA data acquisition, and the scanning range is from 100 to 1700 m/z for MS1. During PASEF MS/MS scanning, the impact energy increases linearly with ion mobility, from 20 eV (1/K0 = 0.6 *Vs*/cm2) to 59 eV (1/K0 = 1.6 *Vs*/cm2).

#### 2.2.2 Proteome discoverer database search

Vendor’s raw MS files were processed using SpectroDive software (11.10.230428.47784) and the built-in Pulsar search engine. MS spectra lists were searched against their species-level UniProt FASTA databases, Carbamidomethyl [C] as a fixed modification, Oxidation (M) and Acetyl (Protein N-term) as variable modifications. Trypsin was used as proteases. A maximum of 2 missed cleavage(s) was allowed. The false discovery rate (FDR) was set to 0.01 for both PSM and peptide levels. Peptide identification was performed with an initial precursor mass deviation of up to 20 ppm and a fragment mass deviation of 20 ppm. All the other parameters were reserved as default.

#### 2.2.3 Establishment of the PRM approach and quantitative analysis

The construction of the PRM method was accomplished through the utilization of SpectroDive software. (1) The DDA data gathered in the previous stage were imported into the target polypeptide sequence columns within SpectroDive software, and the library was searched to generate the corresponding one. (2) Based on the library and target peptide generation, panel screens peptides and confirms quantitative secondary b/y ions. For each target, 3 to 6 fragment ions were chosen for the quantification of polypeptides.

The raw PRM data was processed by means of the Skyline bioinformatics tool (MacCoss Laboratory, University of Washington, USA). Here, the detection signal strength of a single peptide sequence for each target protein was normalized with standard reference and quantified in relation to the corresponding sample. The quantitative file encompasses the outcomes of all selected samples, measuring target proteins, including 13 target proteins and 25 unique peptide sequences. The median log2(A/B) ratio of the peptide conversion was employed to quantify the peptide and acquire the protein log2(A/B) ratio.

### 2.3 Statistical analysis

Baseline demographic and clinical characteristics were compared between groups to confirm balance. Normality of continuous variables was assessed using the Shapiro–Wilk test. For normally distributed variables, comparisons between groups were conducted using the independent-samples Student’s t-test; for non-normally distributed variables, the Mann–Whitney U test was applied. Categorical variables such as gender and eye laterality were compared using the χ^2^ test or Fisher’s exact test when expected counts were small. A two-sided *p*-value < 0.05 was considered statistically significant.

This was a discovery-oriented, two-phase study in which the number of stromal lenticule samples was constrained by the availability of eligible SMILE procedures and by reagent cost; a formal *a priori* power calculation was therefore not performed. The discovery phase (10 eyes per group) was intended to detect proteins with the relatively large between-group differences typical of label-free discovery experiments, and the targeted parallel reaction monitoring (PRM) phase used independent cohorts (15 eyes per group) to verify the prioritized candidates. We recognize that this design may have been underpowered to detect proteins with modest effect sizes, and discovery-phase findings that were not confirmed by PRM are interpreted as hypothesis-generating accordingly (see Limitations).

## 3 Results

### 3.1 Phase I: 4D label-free quantitative proteomic assay

In group A (the compound myopic astigmatism group cohort), there were 9 patients (10 eyes) with an average age of (23.8 ± 9.1) years, including 8 males and 2 females, and having a spherical equivalent refraction of (−5.04 ± 1.80) D, OD = 5/OS = 5. Group B (the simple myopia group) consisted of 10 eyes (OD = 5/OS = 5) from a total of 8 subjects (7 males and 3 females), with an average age of (28.1 ± 7.0) years and a spherical equivalent refraction of (−5.13 ± 2.11) D. No statistically significant differences were observed between the two groups in terms of age, gender distribution, spherical equivalent refraction, or eye laterality preference (*p* > 0.05).

**Figure 1:**
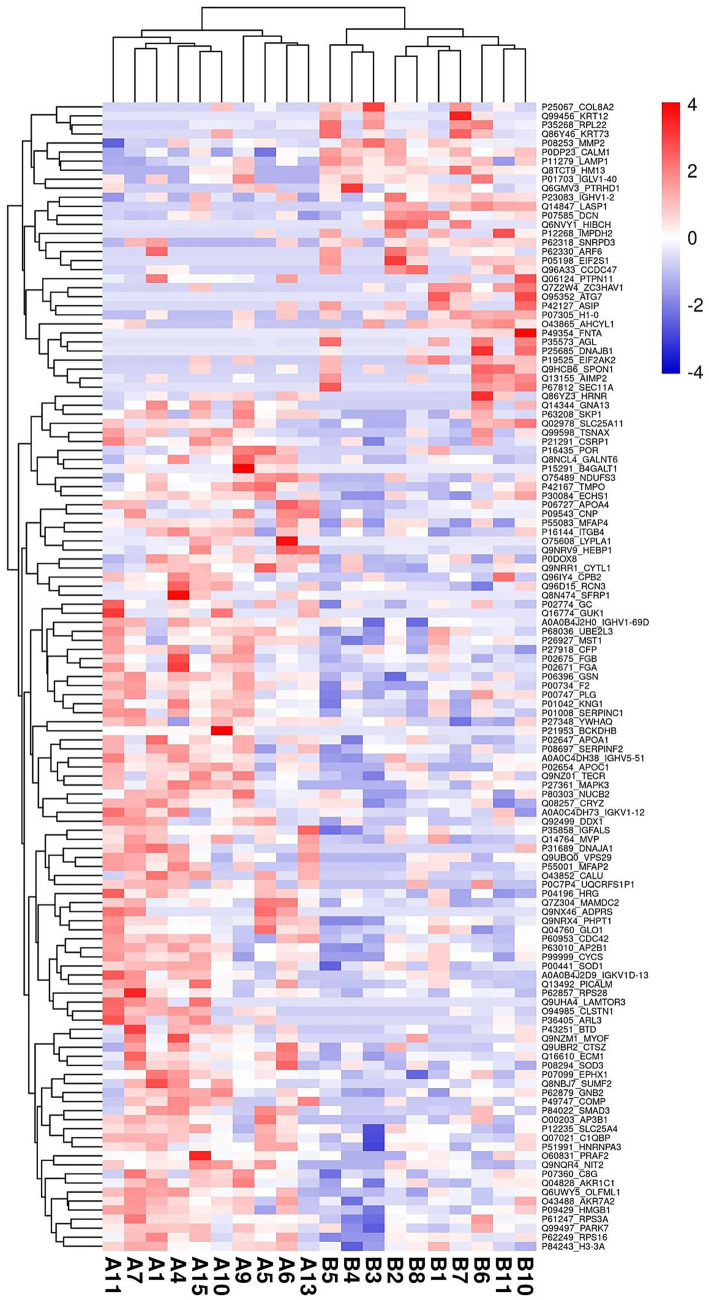
Cluster analysis of DEPs. Hierarchical cluster analysis was executed through the utilization of the tree heat map. In the heatmap, each row embodies a protein (that is, the ordinate represents significantly DEPs), and each column constitutes a set of samples (the abscissa represents sample information). The logarithmic values of significant DEPs (log2 expression) are delineated in diverse colors, with red denoting a significantly up-regulated protein, blue denoting a significantly down-Regulated protein, and gray indicating the non-availability of quantitative protein information.

This research resulted in the identification of a total of 1952 protein groups and 13,581 peptides in the protein atlas of *Homo sapiens* (Table 1). A comparison between compound myopic astigmatism and simple myopia disclosed a total of 127 DEPs, with 31 proteins showing elevated levels and 96 proteins presenting decreased levels (see Supplementary File 2). Subsequently, a heat map was generated to illustrate the hierarchical clustering analysis of the identified DEPs (Figure 1).

**Table 1:** Top five ontology terms for GO and KEGG analysis.

Ontology	Description	Adjust *p*-value	Rich factor
BP	Negative regulation of hemostasis	0.000000598766	26.9835739173718
BP	Fibrinolysis	0.000003545325	38.0879253236977
BP	Negative regulation of blood coagulation	0.0000047271	24.4850948509485
BP	Regulation of hemostasis	0.0000047271	14.9908743985399
BP	Negative regulation of wound healing	0.00000551495	17.6292682926829
CC	Extracellular region	1.38414E-23	2.96966704416761
CC	Extracellular space	7.8234E-23	3.41338277511962
CC	Extracellular region part	4.68066666666667E-22	3.24504908835905
CC	Extracellular exosome	6.5195E-22	4.33157163255593
CC	Extracellular vesicle	7.68966666666667E-22	4.28466666666667
MF	Structural molecule activity	0.0033544	3.54165471066475
MF	Extracellular matrix structural constituent	0.0033544	8.31093820140655
MF	Glycosaminoglycan binding	0.0067088	6.21984814889471
MF	Phosphatidylcholine-sterol O-acyltransferase activator activity	0.0067088	75.260162601626
MF	Heparin binding	0.0201264	6.9204747219886
KEGG	Complement and coagulation cascades	0.0000178430662132824	11.3645863997645
KEGG	Coronavirus disease	0.00556162933168087	4.6808162374509
KEGG	Influenza A	0.0608726341185941	4.4454067658598
KEGG	Regulation of actin cytoskeleton	0.0608726341185941	3.79370957934885
KEGG	Vitamin digestion and absorption	0.0608726341185941	13.5743670886076

GO, gene ontology; KEGG, Kyoto Encyclopedia of Genes and Genomes; BP, biological process; MF, molecular function; CC, cellular component. Group comparisons were conducted using Welch’s t-tests with Benjamini–Hochberg FDR correction.

### 3.2 Phase II: PRM validation study—validation of corneal astigmatism-associated protein

There were 13 patients (15 eyes) with an average age of (21.1 ± 3.2) years, including 10 males and 3 females, and having a spherical equivalent refraction of (−5.38 ± 1.98) D in group A (OD = 8/OS = 7). Group B consisted of 15 eyes (OD = 8/OS = 7) from a total of 14 subjects (10 males and 4 females), with an average age of (23.6 ± 4.4) years and a spherical equivalent refraction of (−4.53 ± 1.55) D. No statistically significant differences were observed between the two groups in terms of age, gender distribution, spherical equivalent refraction, or eye laterality preference (*p* > 0.05).

**Figure 2:**
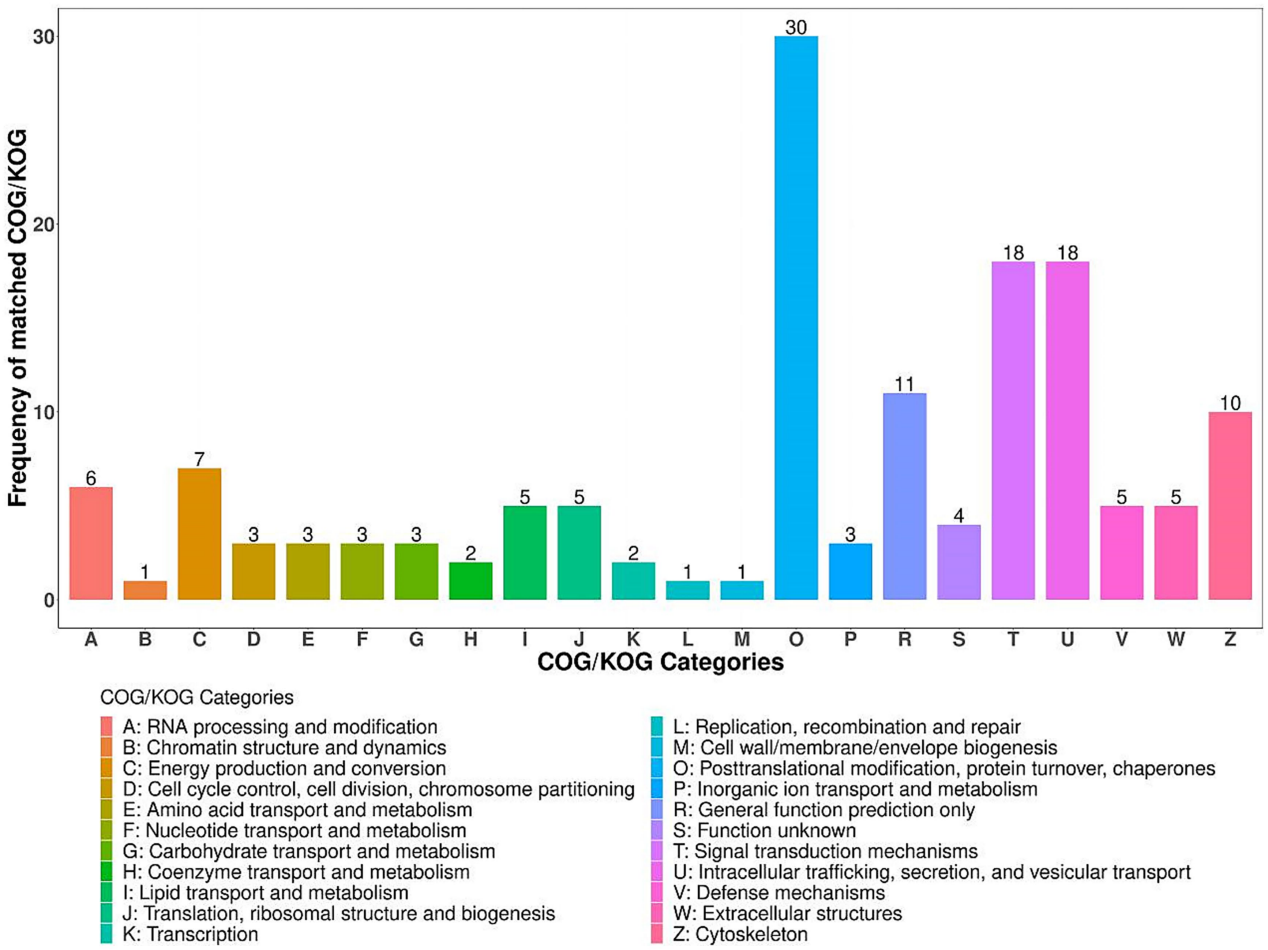
The results of the cluster of orthologous groups of proteins (COG) analysis of DEPs were presented in the form of bar plots. The horizontal coordinate indicates the diverse classification contents of COG, and the vertical coordinate represents the COG frequency. In various functional categories, the proportion of proteins reflects the metabolic or physiological bias in the corresponding period and environment.

**Figure 3:**
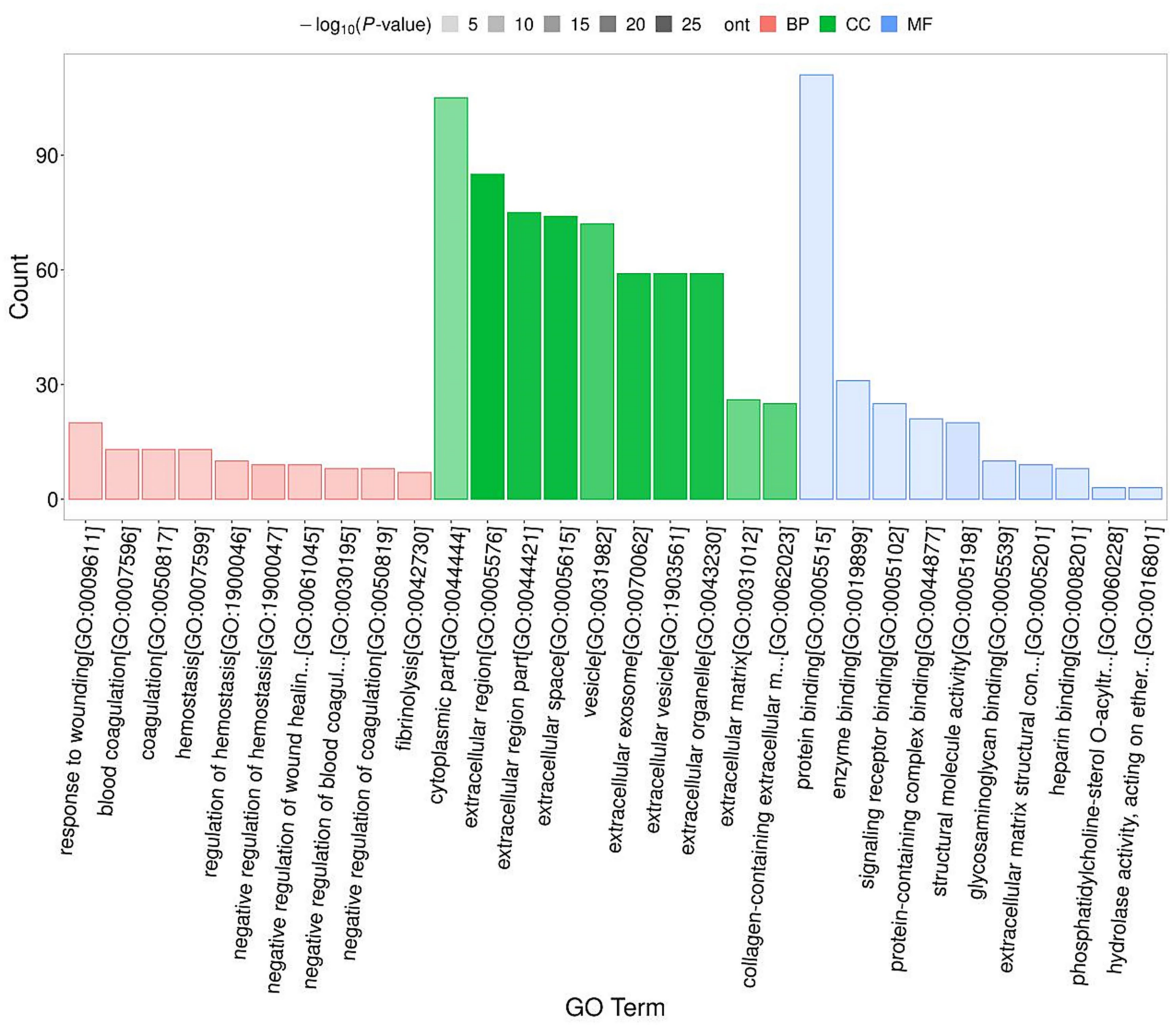
Classification histogram of top 10 GO enrichment analysis of DEPs (A vs. B). In this figure, the horizontal coordinate represents GO Term, and the vertical coordinate indicates the number of differentially expressed proteins in the map. Red symbolizes the biological process (BP) annotation information, green represents the cellular component (CC) annotation information, blue indicates the molecular function (MF) annotation information, and transparency reflects the magnitude of the *p*-value. The darker the color, the smaller the *p*-value. A: myopic astigmatism group; B: simple myopic group.

**Figure 4:**
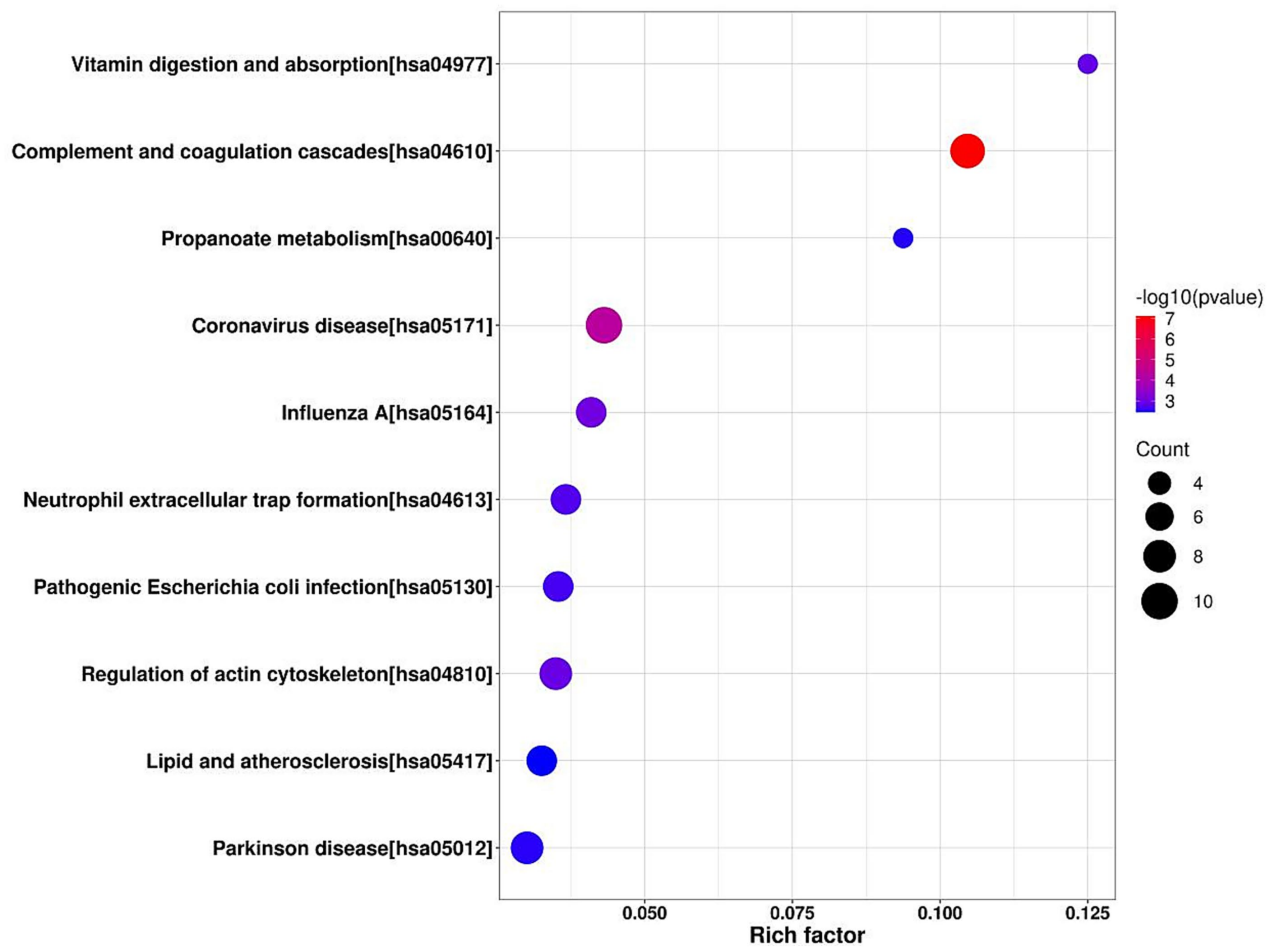
Bubble plot of KEGG pathway enrichment analysis of DEPs (A vs. B). The abscissa represents the Rich factor value, indicating the degree of enrichment, while the y-axis displays KEGG Pathway information. The size of each circle corresponds to the number of differentially expressed proteins in the respective pathway, with larger circles representing a higher count. Additionally, the color of each circle reflects the significance level as indicated by the *p*-value, with redder shades denoting smaller *p*-values. A: myopic astigmatism group; B: simple myopic group.

**Figure 5:**
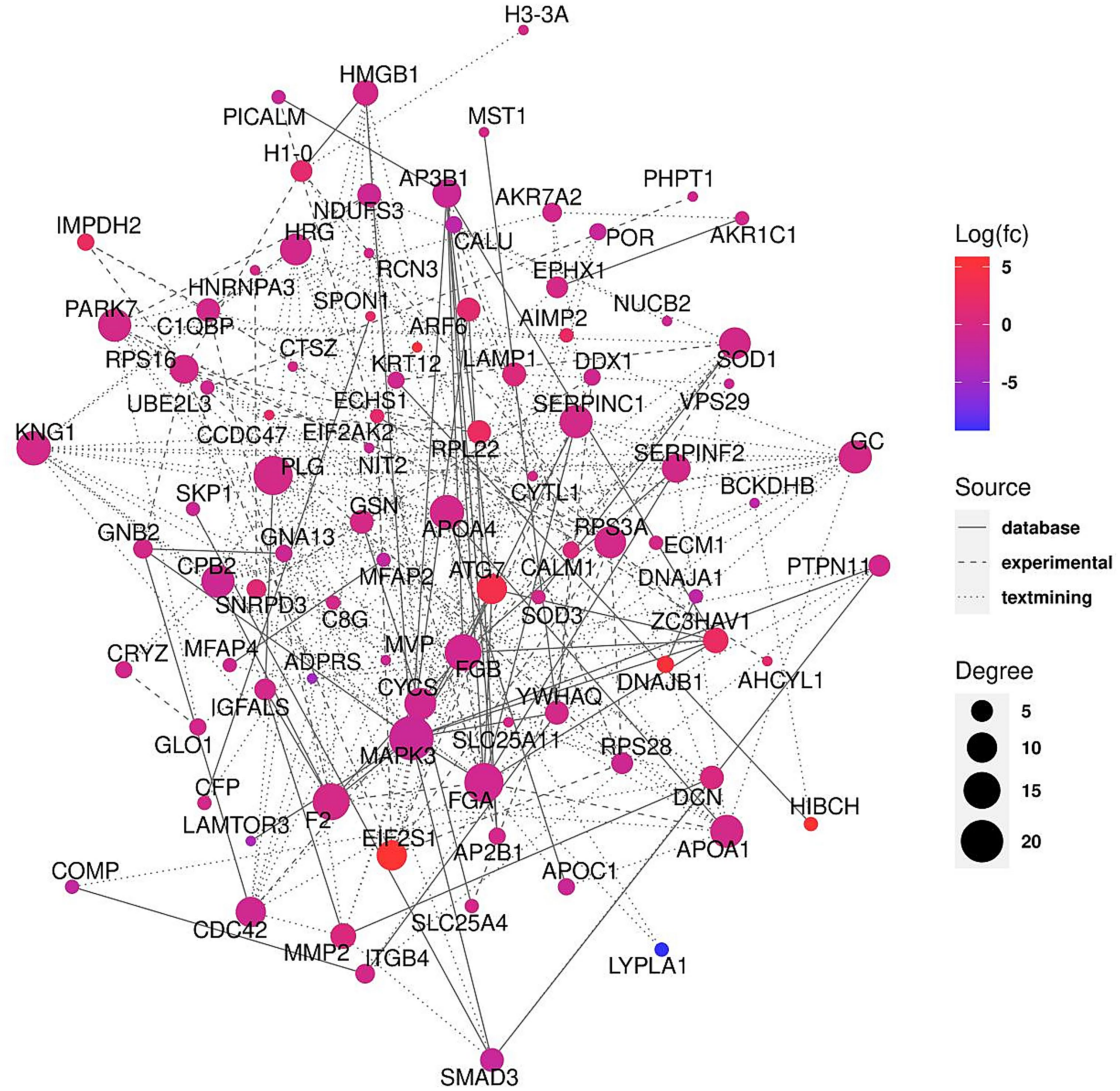
A protein–protein interaction (PPI) network diagram (A vs. B). The colors presented in the figure signify the expression levels of differentially expressed proteins. Specifically, red represents a significantly upregulated expression, while blue indicates a significantly downregulated expression. The size of the circles is an indicator of the degree of connectivity of the differentially expressed proteins, with a larger circle suggesting a higher degree of connectivity. The types of lines reflect the sources of the interaction. A solid line indicates an interaction derived from the database, a dashed line represents an interaction obtained from experiments, and a dotted line depicts an interaction discovered through text mining. A: myopic astigmatism group; B: simple myopic group.

**Figure 6:**
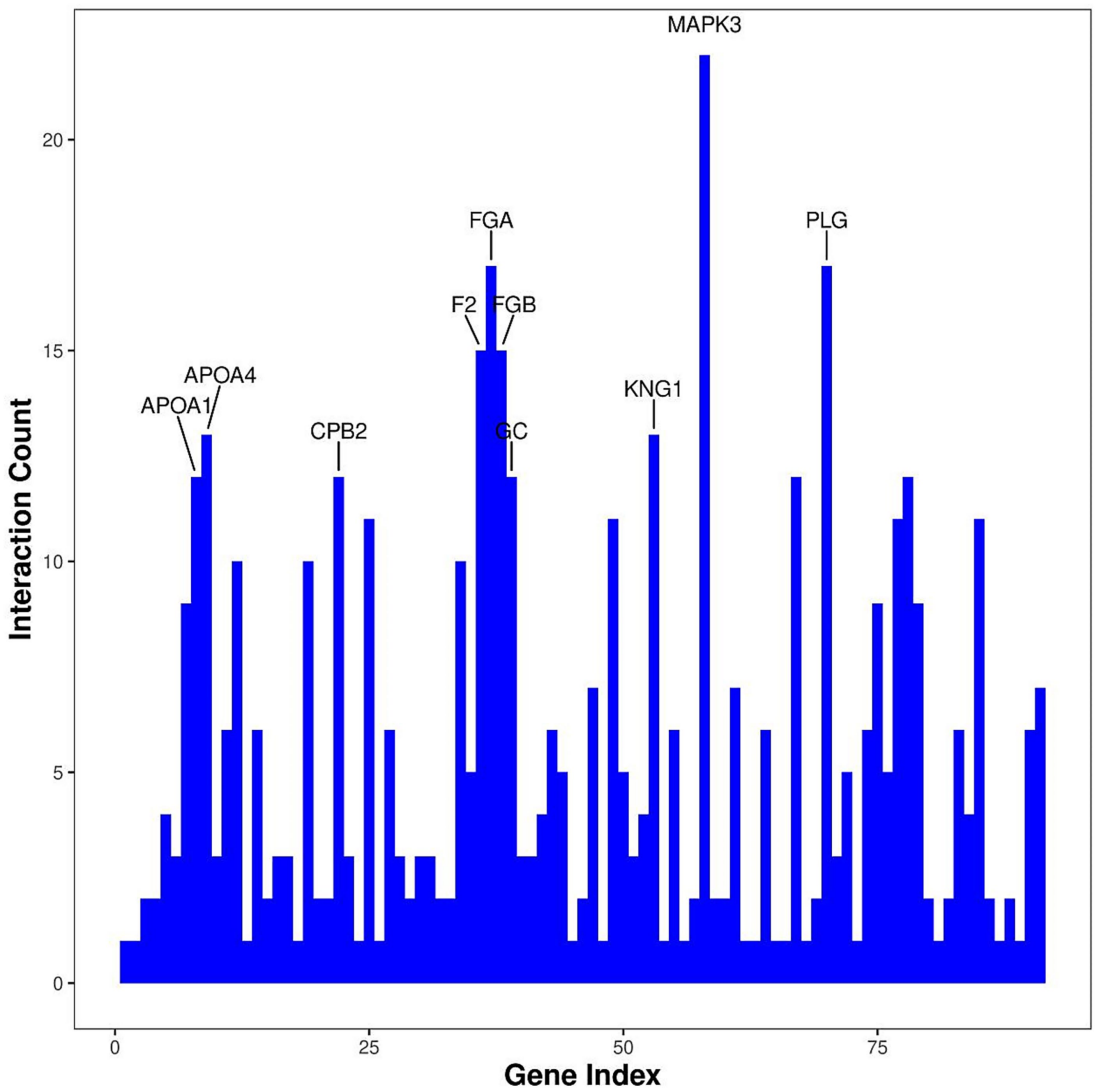
Histogram of connectivity between A and B proteins (A vs. B). The horizontal coordinate denotes the gene index, and the vertical coordinate indicates the interaction count between proteins. A: myopic astigmatism group; B: simple myopic group.

Biomarker Candidates Based on the previous GO annotation (Figures 2, 3), KEGG pathway analysis (Figure 4), and PPI network analysis outcomes (Figure 5), we concentrated on the major GO terms “coagulation” and “trauma response,” as well as the major KEGG pathways “complement and coagulation cascade” and “lipid and atherosclerosis.” Guided by discovery-phase bioinformatics (GO terms related to coagulation/trauma response; KEGG pathways “complement and coagulation cascade” and “lipid and atherosclerosis”; and PPI network features), we prioritized 20 DEPs for targeted quantification by parallel reaction monitoring (PRM). A total of 13 DEPs in the two groups demonstrated a tendency of aggregability, among which 9 proteins related to the “complement and coagulation cascade” and “lipid and atherosclerosis” pathway were verified. Of the targeted panel, only apolipoprotein A-IV (APOA4; P06727) demonstrated a statistically significant decrease in the astigmatism group compared with simple myopia after multiplicity adjustment. Eight additional proteins showed directionally concordant changes with the discovery LFQ results but did not reach statistical significance on PRM; these included KNG1 (P01042), SERPINC1 (P01008), PLG (P00747), FGA (P02671), FGB (P02675), F2 (P00734), APOA1 (P02647), which trended downward, and MMP2 (P08253), which trended upward (see Figure 6 and Table 2). These targets are therefore considered hypothesis-generating and warrant validation in larger, adequately powered cohorts.

**Table 2:** The results of the differential expression of polypeptides between groups were analyzed through PRM.

GeneName	Accession	Sequence	*p*-value	Fold Change	Log_foldChange
SOD1	P00441	_GDGPVQGIINFEQK_	0.061215511	0.82946512	−0.269746779
SOD1	P00441	_TLVVHEK_	0.251903656	1.607046351	0.68441154
F2	P00734	_TATSEYQTFFNPR_	0.635263236	0.957939016	−0.06199428
F2	P00734	_SPQELLC[Carbamidomethyl (C)]GASLISDR_	0.505890704	1.125886911	0.171061924
PLG	P00747	_HSIFTPETNPR_	0.552365229	1.067254191	0.093903827
PLG	P00747	_C[Carbamidomethyl (C)]EEDEEFTC[Carbamidomethyl (C)]R_	0.500087786	0.674309084	−0.568518062
SERPINC1	P01008	_LPGIVAEGR_	0.3853794	0.920363548	−0.119724249
SERPINC1	P01008	_TSDQIHFFFAK_	0.578016471	0.945412175	−0.080984651
KNG1	P01042	_DIPTNSPELEETLTHTITK_	0.623201835	0.93838369	−0.091750157
KNG1	P01042	_AATGEC[Carbamidomethyl (C)]TATVGK_	0.203395366	1.706741914	0.771244917
APOA1	P02647	_THLAPYSDELR_	0.524404917	0.931454882	−0.102442205
APOA1	P02647	_ATEHLSTLSEK_	0.323295063	0.881860545	−0.181377565
FGA	P02671	_GLIDEVNQDFTNR_	0.553193348	1.092771031	0.127991144
FGA	P02671	_VQHIQLLQK_	0.321747143	1.147502121	0.198496821
FGB	P02675	_QGFGNVATNTDGK_	0.574173997	1.076230691	0.105987354
FGB	P02675	_YQISVNK_	0.774736685	1.051322063	0.072204693
GC	P02774	_HLSLLTTLSNR_	0.72134248	0.959385381	−0.059817638
GC	P02774	_LPDATPTELAK_	0.459396683	0.803938241	−0.314843418
EIF2S1	P05198	_VVTDTDETELAR_	0.958763144	0.995259287	−0.006855666
APOA4	P06727	_SLAPYAQDTQEK_	0.045801542*	0.666400734	−0.585538104
APOA4	P06727	_LGEVNTYAGDLQK_	0.040379991*	0.754505617	−0.406396453
MMP2	P08253	_QDIVFDGIAQIR_	0.880197906	1.029758365	0.042305845
MMP2	P08253	_AFQVWSDVTPLR_	0.197390243	0.737167391	−0.43993584
CDC42	P60953	_NVFDEAILAALEPPEPK_	0.728417981	1.038665372	0.054730935
CDC42	P60953	_YVEC[Carbamidomethyl (C)]SALTQK_	0.335239223	0.788723062	−0.342409267

*p < 0.05. Group comparisons were conducted using Welch’s t-tests with Benjamini–Hochberg FDR correction.

## 4 Discussion

### 4.1 Bioinformatic overview and discovery proteomics

**Figure 7:**
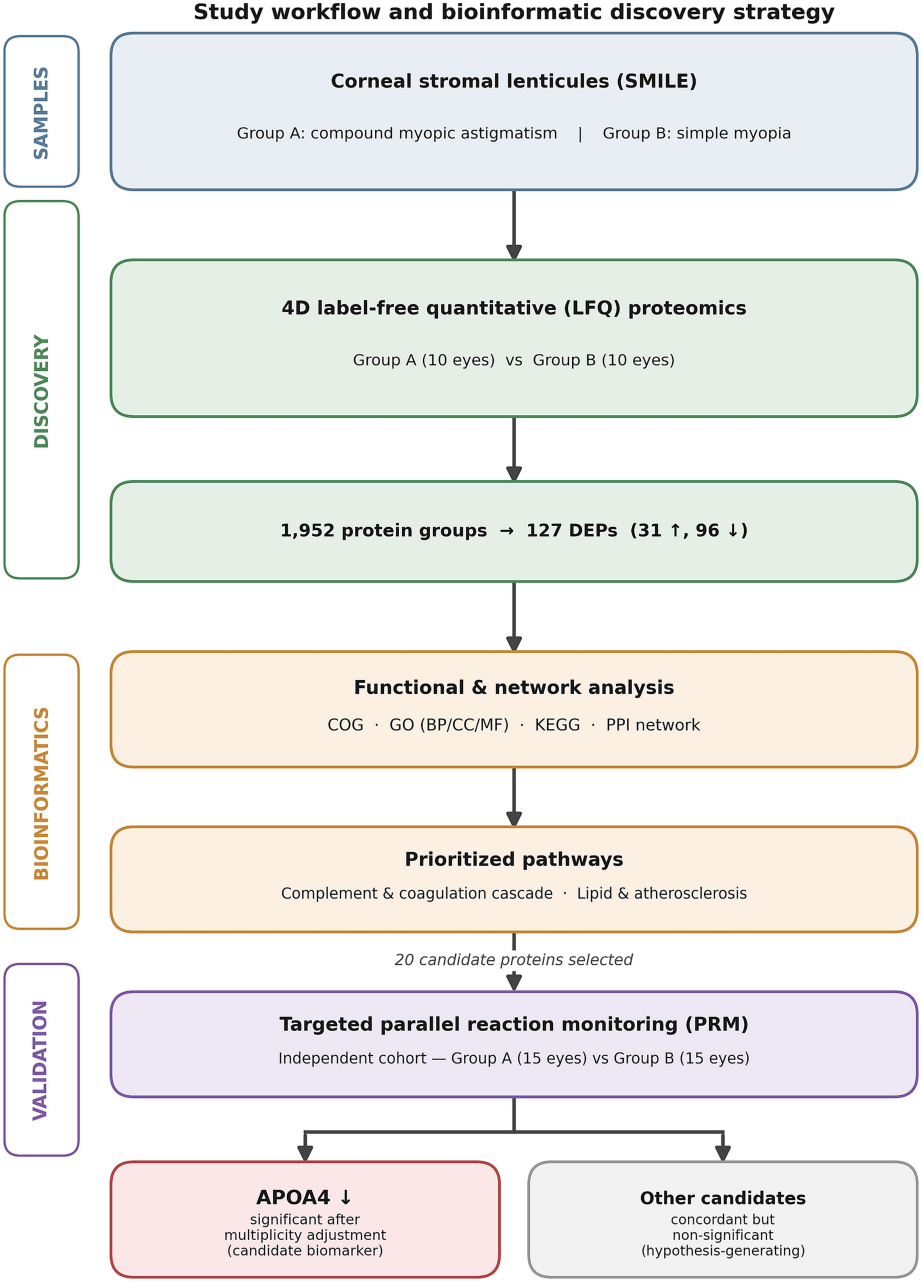
Schematic overview of the study workflow and bioinformatic discovery strategy. Corneal stromal lenticules obtained during SMILE were compared between compound myopic astigmatism (Group A) and simple myopia (Group B). In the discovery phase, 4D label-free quantitative (LFQ) proteomics identified 127 differentially expressed proteins (DEPs; 31 upregulated, 96 downregulated). Bioinformatic analysis (COG, GO, KEGG, and protein–protein interaction [PPI] network mapping) prioritized candidates within the “complement and coagulation cascade” and “lipid and atherosclerosis” processes, from which 20 proteins were selected for targeted parallel reaction monitoring (PRM) in an independent cohort. After multiplicity adjustment, only apolipoprotein A-IV (APOA4) remained significantly reduced in corneal astigmatism; the remaining candidates showed concordant but non-significant trends and are considered hypothesis-generating.

Using label-free quantification, we identified 127 DEPs between compound myopic astigmatism and simple myopia. Functional annotation converged on processes relevant to immune–coagulation, lipid handling/atherogenesis, extracellular matrix (ECM) organization, and oxidative stress regulation. The COG classification highlighted shifts in proteins involved in post-translational modification, cytoskeletal architecture, and lipid metabolism (see Figure 2). GO enrichment (BP/CC/MF) further implicated complement activation, fibrin formation, lipoprotein particle biology, collagen-containing ECM, and antioxidant activity (see Figure 3). KEGG pathway analysis prioritized the complement and coagulation cascade and lipid and atherosclerosis (see Figure 4). Finally, the PPI network revealed a set of high-degree nodes consistent with pathway hubs (see Figures 5, 6). Collectively, these analyses suggest that stromal protein changes in astigmatism converge on inflammation/coagulation–lipid–ECM axes that could influence corneal stromal homeostasis and biomechanics. Because these enrichments derive from discovery-phase label-free data, they were treated as hypotheses to be tested by targeted verification rather than as established mechanisms. The overall analytical workflow—from discovery proteomics and bioinformatic prioritization to targeted PRM verification—is summarized in Figure 7.

### 4.2 Biological processes and cellular components

Gene Ontology (GO) enrichment analysis highlighted that DEPs were heavily involved in wound healing, blood coagulation, and regulation of hemostasis, all processes integral to corneal physiology and healing post-surgery. Representative proteins contributing to these terms included coagulation- and complement-related factors such as KNG1, SERPINC1, PLG, FGA, and FGB (see pathway mapping in Supplementary Files 3–5), consistent with the role of the cornea as a highly regenerative tissue and suggesting that dysregulation of repair/hemostatic programs may contribute to astigmatic aberrations in myopic eyes.

The findings of enriched cellular components such as collagen-containing extracellular matrix and cytoplasmic part may reflect the critical role of structural proteins in maintaining corneal shape and integrity. Given that corneal biomechanics directly influence refractive outcomes, including astigmatism, these findings underscore the importance of extracellular matrix proteins and cytoskeletal components in modulating corneal curvature and rigidity, potentially contributing to the development of astigmatism. In this context, ECM/adhesion-related contributors (e.g., MMP2 and matrix/adhesion proteins listed in Supplementary Files 3–5) align with the PPI hubs observed in Figures 5, 6 and support a remodeling-oriented mechanism.

### 4.3 Complement and coagulation cascades

Among the pathways significantly enriched in the differentially expressed protein dataset, the complement and coagulation cascades pathway (KEGG: Figure 4) was notably implicated in the compound myopic astigmatism group. This finding aligns with previous studies that have linked abnormal complement activation to ocular diseases, including keratoconus (15) and glaucoma (16–18). Although the complement and coagulation systems participate in inflammation and tissue remodeling, the present data are derived from discovery-phase enrichment analysis and do not establish a causal role for these pathways in astigmatism; notably, this signal was not confirmed at the protein level on targeted verification (see below).

Moreover, proteins such as KNG1 (P01042), SERPINC1 (P01008), PLG (P00747), and FGA (P02671), which are key mediators of coagulation, were downregulated in the astigmatic group. These proteins are well-established regulators of fibrin formation and degradation, critical in tissue injury repair processes (19). Downregulation of these coagulation components may suggest impaired wound healing dynamics, potentially resulting in abnormal corneal remodeling and contributing to the persistence or progression of astigmatism after surgery. We note that in targeted verification these factors showed directionally concordant trends but did not reach statistical significance (see PRM section below), and thus are considered hypothesis-generating candidates pending validation.

### 4.4 Lipid metabolism and atherosclerosis pathways

In addition to complement and coagulation cascades, the pathway analysis revealed significant enrichment in proteins involved in lipid metabolism and atherosclerosis (KEGG: Figure 4). In particular, apolipoprotein A-IV (APOA4) was markedly downregulated in the astigmatic group (20). APOA4 has been implicated in a wide array of systemic and ocular disorders, ranging from cardiovascular disease (21) to age-related macular degeneration (22). The reduced levels of APOA4 may contribute to the disrupted lipid metabolism within the corneal tissue, potentially influencing the biomechanical properties of the cornea and exacerbating astigmatic refractive errors. Other lipid-handling proteins observed in discovery (e.g., APOA1; see Supplementary Files 3–5) were directionally consistent with a lipid/atherosclerosis signal, further supporting a lipid-centric axis in corneal stromal homeostasis.

The involvement of lipid and atherosclerosis-related pathways in corneal astigmatism is a novel finding in ophthalmology and warrants further exploration (23). Dysregulated lipid metabolism may affect the composition of cell membranes and the extracellular matrix, altering corneal cell function and structure (24). These observations provide a biologically coherent backdrop for targeted verification of lipid-related candidates, particularly APOA4.

### 4.5 Targeted verification and emphasis on APOA4

In the second phase (PRM), 20 discovery-prioritized proteins were targeted based on pathway relevance and network features. After multiplicity adjustment, only APOA4 demonstrated a statistically significant decrease in the astigmatism group, whereas other candidates—including KNG1, SERPINC1, PLG, FGA, FGB, F2, APOA1, and MMP2—showed directionally concordant but non-significant differences on PRM (see Figure 6 and Table 2). Accordingly, we restrict the term “validated” to APOA4; the remaining targets should be regarded as hypothesis-generating and require confirmation in larger cohorts. The significant downregulation of APOA4 in the astigmatism group provides a specific target for further research. As APOA4 plays a known role in lipid transport and metabolism, its dysregulation in astigmatism suggests a potential biomarker or therapeutic target for future interventions aimed at optimizing corneal healing and refractive outcomes post-surgery.

Several mechanisms could plausibly link reduced stromal APOA4 to corneal biology. APOA4 is a lipid-binding apolipoprotein involved in lipid transport, reverse cholesterol transport, and modulation of lipid-membrane composition; a local reduction could alter the lipid composition of keratocyte membranes and of the stromal extracellular matrix, with downstream effects on cell signaling and matrix organization (25, 26). APOA4 also has reported antioxidant and anti-inflammatory properties and can limit lipid peroxidation (27); lower APOA4 might therefore reduce local antioxidant capacity and increase oxidative stress, a process previously implicated in corneal remodeling and ectatic change (28). Through these lipid-handling and redox-buffering roles, diminished APOA4 could in principle affect keratocyte function, collagen and ECM homeostasis, and stromal biomechanical stability, any of which may influence corneal curvature and astigmatism. We emphasize, however, that these mechanistic links are hypotheses suggested by our data and supported only by indirect evidence; direct functional studies (for example, in keratocyte or corneal-organoid models) are required to establish whether APOA4 causally modulates corneal biomechanics.

While our findings offer valuable insights, they also raise several questions for future exploration. It is not yet clear whether the observed protein dysregulation is a cause or consequence of astigmatism, or whether the differences in protein expression could predict postoperative refractive outcomes in broader patient populations. Additionally, the roles of MMP2 and other matrix metalloproteinases, which were upregulated in the astigmatism group, deserve further investigation in terms of their involvement in corneal remodeling and astigmatism progression. Future studies integrating proteomics with genomics and environmental exposures could help disentangle causal pathways and test whether lipid-centric signals (APOA4) intersect with coagulation/inflammation and ECM remodeling in shaping corneal biomechanics.

### 4.6 Limitations and future directions

This study has several strengths, including the use of advanced proteomics techniques and independent, orthogonal targeted verification (PRM) of discovery-prioritized candidates. However, certain limitations must be acknowledged. The relatively small sample size, although adequate for initial discovery, limits the generalizability of our findings. Additionally, while our study identified key DEPs and pathways, functional assays are necessary to elucidate the exact mechanisms through which these proteins influence corneal structure and refractive outcomes. Furthermore, PRM verification may have been underpowered to detect modest effects across multiple targets; consequently, only APOA4 met significance after multiplicity control, while other proteins remain hypothesis-generating. Finally, detailed medical histories and comorbidity data were not available in this pilot cohort, which limits adjustment for potential confounding and should be addressed in future work. Validation relied on targeted mass spectrometry (PRM) rather than orthogonal antibody-based assays; the small amount of stromal lenticule tissue available precluded western blotting. Although PRM provides quantitatively reliable, peptide-level confirmation, antibody-based validation of APOA4 (for example, western blotting or immunohistochemistry) in larger, independent cohorts would further strengthen confidence in this finding. In addition, ocular surface parameters were not systematically recorded: tear film break-up time (TBUT) and a formal dry eye disease assessment were not available. Because dry eye disease can influence measured astigmatism (29), the absence of these data is a further limitation that should be addressed in future studies.

Future studies should investigate the interplay between genetic susceptibility loci and environmental exposures (e.g., visual habits, metabolic profiles, ocular surface health) in relation to corneal astigmatism. Integration of genomic, proteomic, and environmental data would provide a more comprehensive understanding of disease mechanisms and potential intervention targets.

## 5 Conclusion

This study provides a proteomic characterization of corneal astigmatism in myopic patients. On targeted verification, reduced apolipoprotein A-IV (APOA4) was the only protein that retained statistical significance, supporting it as a candidate biomarker of corneal astigmatism. The discovery-phase associations with wound healing, coagulation, and lipid metabolism pathways were not confirmed at the protein level and should be regarded as hypothesis-generating. Independent validation in larger, adequately powered cohorts, together with functional studies, is needed to confirm these findings and to clarify the biological role of APOA4 in the cornea.

## Data Availability

The raw data supporting the conclusions of this article will be made available by the authors, without undue reservation.
