# Supplementary material for: Proteomic analysis of corneal astigmatism identifies reduced apolipoprotein A-IV as a candidate biomarker
Source: Front Med (Lausanne). 2026 Jul 15;13:1761572. doi: 10.3389/fmed.2026.1761572 (PMC13416920; doi:10.3389/fmed.2026.1761572)
Supplement: Supplementary file 1 [file Table_1.DOCX]

**Sample preparation and nanoLC-MS/MS analysis**

The sample was added with 100 𝜇L RIPA working solution [25 mM Tris-HCl pH 7.6, 15 mM NaCl, 1% NP40, 1% sodium deoxycholate - 1% SDS, Protease inhibitors stock solution (25 x)] thoroughly incorporated, Samples were grinded with steel balls at a low temperature of 35HZ, 4 min, at 12000 rpm, centrifuged at 4℃ for 10 min, and sonicated with cell breaker in ice water bath for 20 min. Then the supernatant was transferred to a new EP tube by centrifugation at 12000 rpm for 10 min at 4 ° C. Protein concentration of samples was determined by BCA method (Beyotime, Shanghai, China).

From each sample, a total of 40 𝜇g of protein were extracted, diluted to approximately 1 ng/ml with RIPA working solution, and the supernatant was eliminated by precipitation with acetone [ANPEL Laboratory Technologies (Shanghai) In] at -20℃. Subsequently, 40 𝜇L of protein resolving solution [100 mM HEPES, 1% SDC (Sigma-Aldrich, St. Louis, USA)] were introduced in an ultrasonic water bath for redissolving the protein, followed by the addition of DTT (Sigma-Aldrich, St. Louis, USA) and IAA (Sigma-Aldrich, St. Louis, USA) for the reduction and alkylation of disulfide bonds. After Trypsin [Promega (Madison, WI, USA)], 2% TFA (Sigma-Aldrich, St. Louis, USA) was utilized to remove the SDC, and the supernatant was extracted into a new EP tube using a high-speed centrifuge at 12,000 rpm. The peptide was desalted by a C18 column demineralizing centrifuge tube in accordance with the protocol, and the supernatant was centrifuged for further mass spectrometry detection.

**nanoLC-MS/MS analysis**

For each sample, 200 ng of total peptides were separated and analyzed with a nano-UPLC (nanoElute2) coupled to a timsTOF Pro2 instrument (Bruker) with a nano-electrospray ion source. Separation was performed using a reversed-phase column (PePSep C18, 1.9 𝜇m, 75 𝜇m × 15 cm, Bruker,Germany). Mobile phases were H2O with 0.1% FA (phase A) and ACN with 0.1% FA (phase B). Separation of sample was executed with a 60 min gradient at 300 nL/min flow rate. Gradient B: 2% for 0 min, 2-22% for 45 min, 22-37% for 5 min, 37-80% for 5 min, 80% for 5 min. The mass spectrometer adopts DDA PASEF mode for DDA data acquisition, and the scanning range is from 100 to 1700 m/z for MS1. During PASEF MS/MS scanning, the impact energy increases linearly with ion mobility, from 20 eV (1/K0 = 0.6 Vs/cm2) to 59 eV (1/K0 = 1.6 Vs/cm2).

**Data analysis**

SpectroMine database search Vendor’s raw MS files were processed using SpectroMine software (4.2.230428.52329) and the built-in Pulsar search engine. MS spectra lists were searched against their species-level UniProt FASTA databases (uniprot_Homo sapiens_9606_reviewed_2023_09. fasta), Carbamidomethyl [C] as a fixed modification, Oxidation (M) and Acetyl (Protein N-term) as variable modifications. Trypsin was used as proteases. A maximum of 2 missed cleavage(s) was allowed. The false discovery rate (FDR) was set to 0.01 for both PSM and peptide levels. Peptide identification was performed with an initial precursor mass deviation of up to 20 ppm and a fragment mass deviation of 20 ppm. All the other parameters were reserved as default.

**Data analysis**

SpectroMine database search Vendor’s raw MS files were processed using SpectroMine software (4.2.230428.52329) and the built-in Pulsar search engine. MS spectra lists were searched against their species-level UniProt FASTA databases (uniprot_Homo sapiens_9606_reviewed_2023_09. fasta), Carbamidomethyl [C] as a fixed modification, Oxidation (M) and Acetyl (Protein N-term) as variable modifications. Trypsin was used as proteases. A maximum of 2 missed cleavage(s) was allowed. The false discovery rate (FDR) was set to 0.01 for both PSM and peptide levels. Peptide identification was performed with an initial precursor mass deviation of up to 20 ppm and a fragment mass deviation of 20 ppm. All the other parameters were reserved as default.

**Bioinformatics analysis**

To conduct a more effective analysis of the data, we undertake a series of data management activities to prepare and organize the raw data. The main steps include: The label-free project conducts data normalization on the original data, and the normalization approach adopted is median value normalization. The number of unique peptide segments of the protein is screened: the number of unique peptide segments is no less than 1. The label-free project simulates Missing Value Recoding from the original data. The numerical simulation method employed is the half of the minimum value method. After pretreatment, 1948 detected proteins were retained.

By employing R (version 3.6.3) and SIMCA software (V16.0.2, Sartorius Stedim Data Analytics AB, Umea, Sweden), the data were logarithmically and centrally processed, followed by principal component analysis. The screening criteria for differentially expressed proteins (DEPs) were a *P*-VALUE < 0.05 in either Student's t test or Chi-square test, along with a FOLD CHANGE（FC）≤ 0.83 or FC ≥ 1.2. Only the DEPs with *P* values less than 0.05 determined by T test and FC conforming to the screening criteria were presented in the form of Volcano Plot. Subsequently, Cluster 3.0 software and Java Treeview software were exploited to rectify and convert the expression values of DEPs in each group as the input for the hierarchical clustering algorithm. In the course of computing the distance matrix, Euclidean distance was employed for distance measurement and Complete Linkage Method was adopted for linkage determination. The heat map of hierarchical cluster analysis was attained.

By conducting a comparative analysis of the identified protein sequence annotation against the COG ([Cluster of Orthologous Groups of proteins](http://www.ncbi.nlm.nih.gov/COG/)), with each cluster of COG encompassing orthologous sequences, the functionality of the sequence can thereby be predicted. Genes were mapped onto nodes in the Homo sapiens (human) database within the framework of Gene Ontology (GO). Fisher's exact probability approach was utilized for the three ontology modules (biological process, molecular features and cell components) and the GO, an enrichment analysis functionality, was leveraged. The Kyoto Encyclopedia of Genes and Genomes, KEGG Pathway database site was explored for the Homo sapiens (human) database to furnish the drawing module for the representation of differentially expressed protein expression content.

The derived *P*-values were further corrected through multiple tests by applying the Benjamin-Hochberg correction (FDR, false discovery rate), and only the GO functional categories and KEGG pathways with *P*-values < 0.05 were regarded as statistically significant. This project established PPI networks of protein interaction information by utilizing a STRING database (v11.5, string-db.org). Cytoscape software (version 3.2.1) was downloaded and the results of DEPs were imported to query the protein interaction in the STRING database Homo sapiens (human). Visualization and functional analysis of the PPI network were conducted, and network interaction maps of DEPs were constructed.
